# Trace amine-associated receptor 1 (TAAR1) agonism for psychosis: a living systematic review and meta-analysis of human and non-human data

**DOI:** 10.12688/wellcomeopenres.21302.1

**Published:** 2024-04-11

**Authors:** Spyridon Siafis, Virginia Chiocchia, Malcolm R. Macleod, Charlotte Austin, Ava Homiar, Francesca Tinsdeall, Claire Friedrich, Fiona J. Ramage, Jaycee Kennett, Nobuyuki Nomura, Olena Maksym, Grazia Rutigliano, Luke J. Vano, Robert A. McCutcheon, David Gilbert, Edoardo G. Ostinelli, Claire Stansfield, Hossein Dehdarirad, Damian Omari Juma, Simonne Wright, Ouma Simple, Olufisayo Elugbadebo, Thomy Tonia, Ioannis Mantas, Oliver D. Howes, Toshi A. Furukawa, Lea Milligan, Carmen Moreno, Julian H. Elliott, Janna Hastings, James Thomas, Susan Michie, Emily S. Sena, Soraya Seedat, Matthias Egger, Jennifer Potts, Andrea Cipriani, Georgia Salanti, Stefan Leucht

**Affiliations:** 1 Department of Psychiatry and Psychotherapy, TUM School of Medicine and Health, Technical University of Munich, Munich, Germany; 2 German Center for Mental Health (DZPG), partner site München/Augsburg, Germany; 3 Institute of Social and Preventive Medicine, University of Bern, Bern, Switzerland; 4 Centre for Clinical Brain Sciences, The University of Edinburgh, Edinburgh, Scotland, UK; 5 Department of Psychiatry, University of Oxford, Oxford, England, UK; 6 Oxford Precision Psychiatry Lab, NIHR Oxford Health Biomedical Research Centre, Oxford, UK; 7 Department of Psychosis Studies, Institute of Psychiatry, Psychology and Neuroscience, King's College London, London, England, UK; 8 Oxford Health NHS Foundation Trust, Warneford Hospital, Oxford, UK; 9 GALENOS Global Experiential Advisory Board, InHealth Associates, London, UK; 10 EPPI Centre, Social Research Institute, University College London, London, England, UK; 11 My Mind Our Humanity, Young Leaders for Global Mental Health, Mombasa, Kenya; 12 Stellenbosch University/South African Medical Research Council Genomics of Brain Disorders Extramural Research Unit, Department of Psychiatry, Stellenbosch University, Stellenbosch, Western Cape, South Africa; 13 Department of Psychiatry, College of Medicine, University of Ibadan, Ibadan, Oyo, Nigeria; 14 Department of Clinical Neuroscience, Karolinska Institutet, Stockholm, Sweden; 15 Institute of Clinical Sciences (ICS), Faculty of Medicine, Imperial College London, London, England, UK; 16 Department of Health Promotion and Human Behavior, Kyoto University Graduate School of Medicine/School of Public Health, Kyoto, Japan; 17 Department of Clinical Epidemiology, Kyoto University Graduate School of Medicine/School of Public Health, Kyoto, Japan; 18 MQ Mental Health Research, London, UK; 19 Department of Child and Adolescent Psychiatry, Institute of Psychiatry and Mental Health, Hospital General Universitario Gregorio Marañón, IiSGM, CIBERSAM, ISCIII, School of Medicine, Universidad Complutense de Madrid, Madrid, Community of Madrid, Spain; 20 Cochrane Australia, School of Public Health and Preventive Medicine, Monash University, Clayton, Victoria, Australia; 21 Future Evidence Foundation, Melbourne, Australia; 22 Institute for Implementation Science in Health Care, University of Zurich, Zurich, Switzerland; 23 School of Medicine, University of St. Gallen, St. Gallen, Switzerland; 24 Centre for Behaviour Change, University College London, London, England, UK

**Keywords:** Antipsychotics, TAAR1, schizophrenia, clinical trials, preclinical studies, meta-analysis, systematic review, living evidence

## Abstract

**Background:**

Trace amine-associated receptor 1 (TAAR1) agonism shows promise for treating psychosis, prompting us to synthesise data from human and non-human studies.

**Methods:**

We co-produced a living systematic review of controlled studies examining TAAR1 agonists in individuals (with or without psychosis/schizophrenia) and relevant animal models. Two independent reviewers identified studies in multiple electronic databases (until 17.11.2023), extracted data, and assessed risk of bias. Primary outcomes were standardised mean differences (SMD) for overall symptoms in human studies and hyperlocomotion in animal models. We also examined adverse events and neurotransmitter signalling. We synthesised data with random-effects meta-analyses.

**Results:**

Nine randomised trials provided data for two TAAR1 agonists (ulotaront and ralmitaront), and 15 animal studies for 10 TAAR1 agonists. Ulotaront and ralmitaront demonstrated few differences compared to placebo in improving overall symptoms in adults with acute schizophrenia (N=4 studies, n=1291 participants; SMD=0.15, 95%CI: -0.05, 0.34), and ralmitaront was less efficacious than risperidone (N=1, n=156, SMD=-0.53, 95%CI: -0.86, -0.20). Large placebo response was observed in ulotaront phase-III trials. Limited evidence suggested a relatively benign side-effect profile for TAAR1 agonists, although nausea and sedation were common after a single dose of ulotaront. In animal studies, TAAR1 agonists improved hyperlocomotion compared to control (N=13 studies, k=41 experiments, SMD=1.01, 95%CI: 0.74, 1.27), but seemed less efficacious compared to dopamine D _2_ receptor antagonists (N=4, k=7, SMD=-0.62, 95%CI: -1.32, 0.08). Limited human and animal data indicated that TAAR1 agonists may regulate presynaptic dopaminergic signalling.

**Conclusions:**

TAAR1 agonists may be less efficacious than dopamine D _2_ receptor antagonists already licensed for schizophrenia. The results are preliminary due to the limited number of drugs examined, lack of longer-term data, publication bias, and assay sensitivity concerns in trials associated with large placebo response. Considering their unique mechanism of action, relatively benign side-effect profile and ongoing drug development, further research is warranted.

**Registration:**

PROSPERO-ID: CRD42023451628.

## Plain language summary

There is a need for more effective treatments for psychosis, including schizophrenia. Psychosis is a collection of mental health symptoms, such as hearing voices, that can cause distress and impair functioning. These symptoms are thought to be caused by changes in a chemical messenger system in the brain called dopamine. Currently used antipsychotic medications target brain receptors that respond to dopamine. They are not effective in some people and can cause uncomfortable adverse events, such as weight gain and movement disorders, especially with long-term use. A new type of drug is the trace amine-associated receptor 1 (TAAR1) agonists. These drugs act on different brain receptors that can affect the activity of the dopamine system, but do not directly bind to dopamine receptors.

We aimed to understand if TAAR1 agonists can reduce symptoms of psychosis, what adverse events they might have, and how they work. We did this by reviewing and collating all available evidence until November 2023. This is a “living” systematic review, so it will be regularly updated in the future.

We looked at both human and animal studies investigating TAAR1 agonists. Human studies suggested that two TAAR1 agonists (namely, ulotaront or ralmitaront) might have little to no effect on reducing symptoms of psychosis compared to placebo in people with schizophrenia. They seemed to cause fewer adverse events than current antipsychotics. Data from animal studies suggested that TAAR1 agonists had some positive effects but potentially smaller than other antipsychotics. There were little to no data from both human and animal studies about how TAAR1 agonists actually work.

From the current evidence we are uncertain about these results. With the ongoing development of new TAAR1 agonists, more evidence is needed to understand their potential role in the treatment of psychosis.

## Background

Antipsychotic medications constitute the cornerstone of treatment in psychosis ^1, 2^. However, they are associated with high failure rates ^3^ and multiple debilitating adverse events ^1^. Consequently, there is a critical need to develop more efficacious and safer medications for psychosis beyond the current antipsychotics that act as dopamine D _2_ receptor antagonists. Despite efforts, drug discovery in psychosis has frequently failed to identify non-dopaminergic medications in recent years, with many drugs that showed promise in animal studies failing in clinical trials, highlighting a translational disconnect ^4, 5^.

Trace amine-associated receptor 1 (TAAR1) agonism is a potentially emerging and novel mechanism proposed for treating psychosis ^6^. Currently, two TAAR1 agonists, ulotaront (SEP-363856, TAAR1 agonist and serotonin 5-HT _1A_ receptor partial agonist) and ralmitaront (RO6889450, TAAR1 partial agonist), have been under clinical development, but recent clinical trials have had inconclusive findings despite showing promise in preclinical studies ^6– 8^. Nonetheless, the volume of data on TAAR1 agonism is rapidly increasing, and additional compounds are undergoing preclinical development ^6, 9– 11^. There remains uncertainty regarding their effects, underlying mechanism of action, and potential role in the treatment of psychosis ^6^.

### Objectives

Therefore, we co-produced a living systematic review and meta-analysis of human and non-human studies to examine the efficacy, tolerability, and underlying mechanism of action of TAAR1 agonism for psychosis. Within the GALENOS project ^12^, we are committed to co-production and believe that including those with lived experience (“experiential advisors”) in developing this review will increase the relevance of findings ^13, 14^. In the first iteration, we focused on evidence from controlled human and non-human studies comparing TAAR1 agonists with placebo conditions or currently licensed antipsychotics.

## Methods

This report refers to the first iteration of a living systematic review conducted within the GALENOS project ^12^. We used the PRISMA statement for reporting the systematic review (PRISMA 2020) ^15^ and searches (PRISMA-S) ^16^ and the GRIPP-2 short form ^17^ for reporting Patient and Public Involvement (checklists in extended data).

The protocol of the living review was published in Wellcome Open Research ^18^, and registered with PROSPERO ( CRD42023451628) and Open Science Framework ( https://doi.org/10.17605/OSF.IO/86Z2P). Adaptations and deviations from protocol, and more detailed descriptions of methods are reported in extended data.

### Living review methodology

We employ a living review approach, as detailed in our protocol ^18^, due to the ongoing development and research on TAAR1 agonists ^6, 9– 11^. We conduct searches and screen new data every three months. However, there was a three-month delay in searching for non-human studies due to the extensive volume of data in the initial search (see “Study identification”). Additionally, we are developing methods to automatise these processes. Review updates will be initiated upon identifying new data that could materially alter previous findings. Independently of the publication of the review findings and with sufficient human and non-human data, a multidisciplinary expert panel reviews the synthesized evidence in a triangulation meeting, and subsequently experiential advisors set future research priorities, as outlined within the GALENOS project ^12^. Decisions regarding the modification of review questions, methods or the cessation of the living mode will be made considering the outcomes of the triangulation meeting and research prioritization exercises.

### Eligibility criteria and outcomes

***Eligibility criteria***

We included human and non-human controlled experimental studies investigating TAAR1 agonists compared to placebo conditions or antipsychotics in individuals (with or without psychosis), and “animal models of psychosis” (i.e., laboratory methods of inducing psychosis-like features and behaviours in animals), respectively. We used broad eligibility criteria enabling a comprehensive and streamlined data synthesis from both human and non-human studies. A more detailed description of study eligibility criteria and outcomes can be found in extended data.

Regarding human studies, we decided *post hoc* to focus on randomised controlled trials (RCTs), but we also considered uncontrolled experimental studies on neurobiological outcomes due to the limited available data from controlled studies.

***Outcomes***

In human studies, the primary outcome was the severity of overall psychosis symptoms measured by validated rating scales, such as the Positive and Negative Syndrome Scale (PANSS) ^19^. Secondary outcomes included severity of symptom domains and response to treatment, dropouts due to any reason and adverse events, serious and specific adverse events frequently reported with psychotropics ^1, 20^. We conducted separate analyses of efficacy outcomes for subpopulations based on diagnosis (e.g., schizophrenia, Parkinson’s Disease psychosis). We pooled the different populations for dropouts and adverse events, as no substantial differences are expected for these outcomes across populations ^21^.

In non-human studies, primary outcomes included increased locomotor activity and impairment of prepulse inhibition of the acoustic startle reflex. These behavioural measures have some predictive validity in detecting antipsychotic effects ^4, 22– 25^. Secondary outcomes included other behavioural measures such as cognitive function, dropouts, and adverse events.

We also examined potential mechanistic insights by presenting narratively the findings from human and non-human studies on measures of neurotransmitter signalling. Moreover, we investigated the effects of TAAR1 agonists compared to control in TAAR1-knockout animals to determine whether observed differences could be attributed to TAAR1 agonism.

### Study identification

The search strategies were reported in extended data. HD and CS conducted searches for human studies (until 17.11.2023) in PubMed/MEDLINE, Embase, International Pharmaceutical Abstracts, Web of Science, Biosis, PsychINFO, CENTRAL, OpenAlex, clinicaltrials.gov, WHO-ICTRP and ScanMedicine. MRM and FR conducted searches for the non-human studies (until 28.08.2023) in PubMed, Scopus, Web of Science, and PsychINFO.

### Study selection, data extraction and risk of bias evaluation

We used similar methods for study selection, data extraction, and risk of bias evaluation of human and non-human studies, which were conducted by two independent reviewers (SS, CA, CF, NN, JK, GR, LV for human studies; FT, FR, CA, CF, JK, OM and MRM for non-human studies) with reconciliation of discrepancies by senior authors (SS, RAM and SL for human studies; MRM for non-human studies). These processes for human studies were conducted in Evidence for Policy, and Practice Information and Coordinating Centre (EPPI-Reviewer) ^26^ and for non-human studies in Systematic Review Facility (SyRF) ^27^.

***Data extraction process***

We extracted data for study characteristics and outcomes. For continuous outcomes, we preferred change over endpoint scores, and data based on methods accounting for missing data. For dichotomous outcomes, we used the number of individuals/animals allocated to a group as denominator. For crossover studies, we opted to use data from the first phase ^28^. However, when only data from the entire trial duration were reported, we applied appropriate methods to correct the variance ^21, 28^, assuming a correlation (i.e., ρ=0.2 for dropouts and adverse events). We extracted data from variations of the same outcome measure in non-human studies and synthesized them jointly (see “Data analysis”).

We extracted and analysed data at three prespecified timepoints: between 3 and 13 weeks (primary timepoint), shorter-term measurements spanning from immediate post-administration of the intervention to 3 weeks, and longer-term measurements beyond 13 weeks.

***Risk of bias assessment***

We evaluated risk of bias for all outcomes using the RoB2 tool for RCTs ^29^ and the SYRCLE’s tool for animal studies ^30^. Moreover, we assessed reporting completeness of animal studies using a modified ARRIVE Essential 10 checklist ^18, 31^.

### Data analysis

Data analysis was conducted separately for human and non-human studies (SS, and MRM, FR, FT, respectively, supervised by VC and GS). We analysed data comparing TAAR1 agonists versus inactive control conditions or antipsychotics in human and non-human studies, and TAAR1 agonists combined with antipsychotics versus antipsychotics alone in non-human studies.

***Effect sizes***

Effect sizes for continuous outcomes were standardised mean differences (SMDs) and mean differences for weight, prolactin levels, and QTc interval, and for dichotomous outcomes were odds ratios (ORs). We also used normalised mean differences (NMD) in sensitivity analysis of non-human studies if there were sufficient data for sham procedures ( extended data). We presented effect sizes with 95% confidence intervals (95%CI).

***Data synthesis approach***

We synthesized data with meta-analysis whenever sufficient data were available from at least two independent effect sizes for the same outcome. Additionally, we presented effect estimates even when available data were only from a single human study. However, we did not consider such data further for non-human studies; the substantial heterogeneity observed in reviewing animal studies means the findings of a single study are of limited value.

We conducted random-effects meta-analysis for human studies using restricted maximum likelihood to estimate the between-study variance (τ ^2^). For non-human studies, we used a multilevel random-effects meta-analysis ^32^ when there were at least 5 unique categories for at least one level. The three nested levels, from higher to lower, were animal strain, study record, and experiment ( extended data). We used t-distributions, considering degrees of freedom for the multilevel model, to adjust the confidence intervals ^33^. To account for correlated sampling errors, we estimated the within-study variance-covariance matrix by assuming a correlation of ρ=0.5 ^32^ and clustering by publication.

***Exploration of heterogeneity***

Heterogeneity was quantified using 95% prediction intervals and, in human studies τ ^2^, and presented when there were data from more than five studies to estimate the heterogeneity variance. For non-human studies, we reported the variance attributable to each of the nested levels. Potential sources of heterogeneity were explored in predefined meta-regressions ^18^. The data were limited for human studies, but we provided pooled estimates for the primary outcome for the different TAAR1 agonists and doses. For non-human studies, we conducted meta-regressions for sex, induction method, drug characteristics, dose, timing of intervention, risk of bias, and reporting completeness ( *post hoc,* extended data).

***Sensitivity analyses***

We examined the robustness of the findings with pre-defined sensitivity analyses, using: 1) fixed-effects meta-analyses of human studies ( *post hoc*, due to small number of studies), 2) NMD in non-human studies, 3) different assumptions for sampling correlations (ρ=0.2/0.8) in non-human studies, and 4) robust variance estimation in non-human studies ( extended data) ^32, 34^.

***Reporting bias***

We assessed reporting bias for all outcomes in human studies using the ROB-ME (risk of bias due to missing evidence) tool ^35^. We examined small-study effects with regression-based tests ( extended data) ^32, 36^, whenever data were available from at least 10 human or non-human studies.

***Summary of the evidence***

We assessed and presented within-study biases, across-study biases, indirectness, and other biases in predefined summary of evidence tables ^18^.

***Software***

Data analysis was conducted in R statistical software v.4.3.1/2 ^37^ using PRISMA2020 v1.1.1 ^38^, robvis v0.3.0.900 ^39^, tidyverse v2.0.0 ^40^, meta v6.5-0 ^41^ (human studies), orchard v2.0 ^42^ and metafor 4.4-0 ^33^ and (non-human studies). The complete list of packages can be found in detailed results reports ( extended data).

### Co-production methods

The review was co-produced by multiple stakeholders, including experiential advisors ^13^. The topic was drawn from previous prioritisation exercises with patient and public involvement (for instance, James Lind Alliance), and experiential advisors were involved in designing the protocol, interpreting the findings, preparing plain language summaries and disseminating the findings to the wider public ^12, 14^.

## Results

The extended data included detailed reports with summary of evidence tables ^43, 44^.

### Findings from human studies

***Study description***

**Figure 1.**
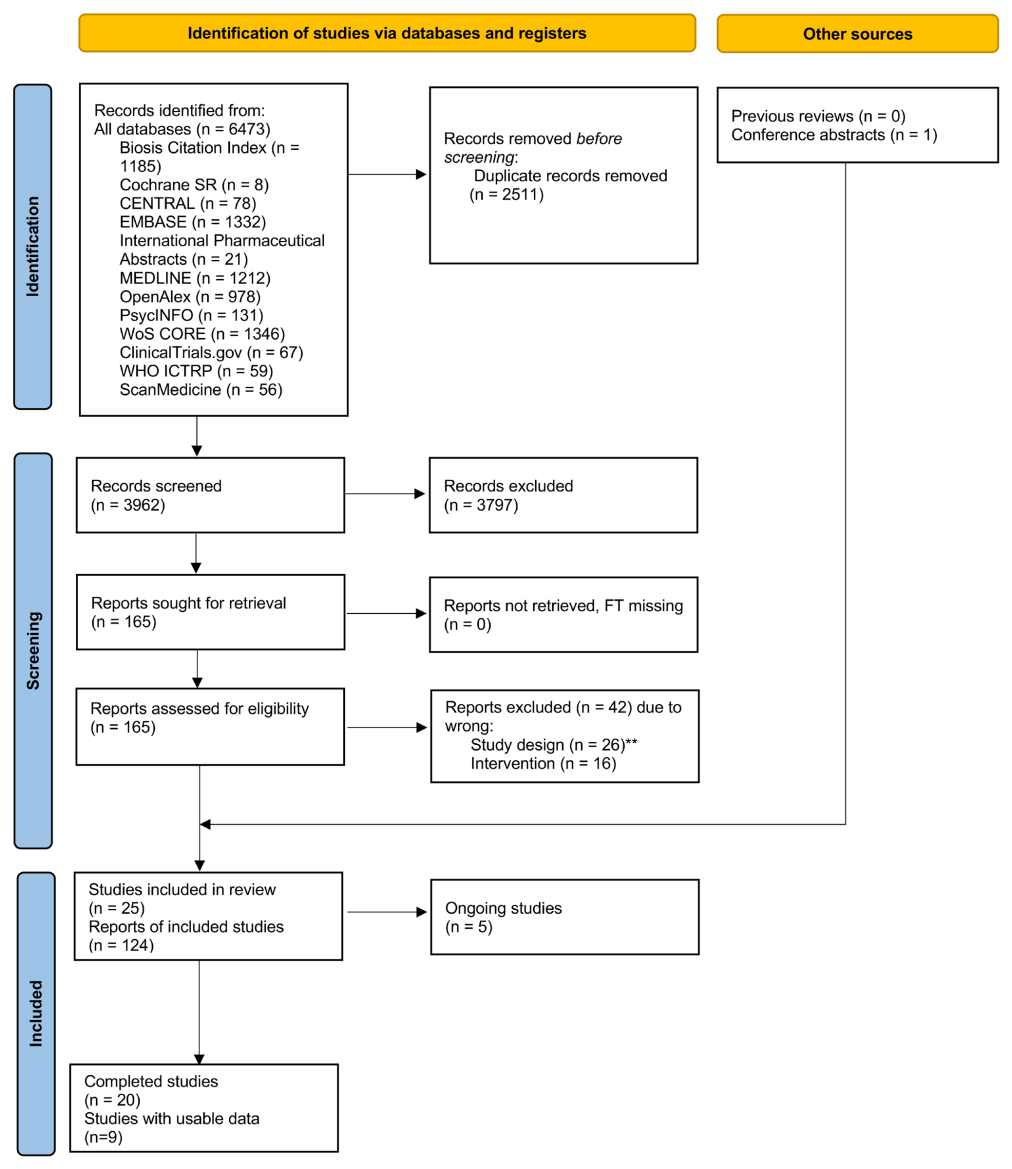
PRISMA flow diagram for study selection of human studies. **One single-arm neuroimaging study (NCT04038957) had originally been listed here as excluded, but its findings were *post-hoc* considered to provide mechanistic insights of the effects of TAAR1 agonists.

We screened 3962 titles/abstracts and 166 full texts ( Figure 1). We included 25 RCTs, 20 completed (2147 participants) and 5 still ongoing on 20.01.2024 ( extended data). There were usable data from 9 RCTs ^7, 45– 52^ with 1683 adult participants (median of mean age 32.9 years, 69.8% men, 30.2% women) conducted by pharmaceutical industries ( Table 1). These trials examined two TAAR1 agonists (ulotaront and ralmitaront). Five studies had parallel design examining the efficacy and tolerability of ulotaront ^7, 45, 50, 51^ or ralmitaront ^52^ compared with placebo (i.e., inert substance) and/or risperidone ^52^ over 4–6 weeks in acute schizophrenia ^7, 50– 52^ or Parkinson’s Disease psychosis ^45^. The other four trials had parallel or crossover design and examined treatment with ulotaront as single dose ^46, 48, 49^ or up to 2 weeks ^47^. Additionally, we considered a single-arm neuroimaging study examining the effects of 2-week treatment of ulotaront on dopamine synthesis capacity in schizophrenia ^53^.

**Table 1.** Table of characteristics of human studies.

Study name	Study ID	Year	Design	Population	Intervention	Sample size	Objectives	Usable data
Hopkins *et al.* 2021 ^49^	SEP361-103	2021	DB-RCT two-period crossover; single dose (phase I)	Men 18-35 years	Cohort 1: Ulotaront 10mg; Placebo Cohort 2: Ulotaront 50mg; Placebo	24	Sleep parameters, pharmacokinetics	Dropouts, side-effects
Isaacson *et al.* 2023 ^45^	SEP361-203, NCT02969369	2023	DB-RCT; 6 weeks (with open label extension) (phase 2)	Men/women ≥55 years with Parkinson's disease psychosis (acute)	Ulotaront 20-75mg/d; Placebo	39	Efficacy (acute), safety	Efficacy, dropouts, side-effects
Koblan *et al.* 2020 ^7^	SEP361-201, NCT02969382, EUCTR2016-001555-41	2018	DB-RCT; 4 weeks (with open label extension) (phase 2)	Men/women 18-40 years with schizophrenia (acute, DSM-5)	Ulotaront (50-75mg/d); Placebo	245	Efficacy (acute), tolerability	Efficacy, dropouts, side-effects
NCT04072354 ^50^	SEP361-301, NCT04072354, EUCTR2019-000470-36	2023	DB-RCT; 6 weeks (phase 3)	Men/women 13-17 and 18-65 years with schizophrenia (acute, DSM-5) **	Ulotaront 50mg/d; Ulotaront 75mg/d; Placebo	463	Efficacy (acute), tolerability	Efficacy, dropouts
NCT04092686 ^51^	SEP361-302, NCT04092686, EUCTR2019-000697-37	2023	DB-RCT; 6 weeks (phase 3)	Men/women 18-65 years with schizophrenia (acute, DSM-5)	Ulotaront 75mg/d; Ulotaront 100mg/d; Placebo	462	Efficacy (acute), tolerability	Efficacy, dropouts
NCT04512066 ^52^	BP41743, NCT04512066, JPRN-jRCT2031200288	2022	DB-RCT; 8 weeks (up to 48 weeks extension) (phase II)	Men/women 18-45 years with schizophrenia or schizoaffective disorder (acute, DSM-5)	Ralmitaront 45mg/d; Ralmitaront 150mg/d; Risperidone 4mg/d; Placebo	287	Efficacy (acute), tolerability	Efficacy, dropouts, side-effects
Perini *et al.* 2023 ^46^	SEP361-104, NCT01972711	2015	DB-RCT; single dose (phase 1)	Men/women 18-45 years (high or low levels of schizotypy)	Ulotaront 50mg; Amisulpride 400mg; Placebo	105	fMRI	Dropouts, side-effects, fMRI (narratively)
Szabo *et al.* 2023 ^47^	SEP361-108, NCT05015673	2015	DB-RCT three-period crossover; 2 weeks (phase 1)	Men/women 18-55 years with narcolepsy/cataplexy	Ulotaront 25mg; Ulotaront 50mg; Placebo	18	Sleep parameters, pharmacokinetics, tolerability	Dropouts, side-effects
Tsukada *et al.* 2023 ^48^	SEP361-114, NCT04369391	2020	DB-RCT three-period crossover; single dose (phase 1)	Men/women 18-65 years with schizophrenia (stable, DSM-5)	Ulotaront 150mg; Placebo; Moxifloxacin 400mg (ineligible for the review)	68	QTc interval, tolerability, pharmacokinetics	Dropouts, side-effects
NCT04038957 * ^53^	SEP361-118, NCT04038957, 2019-000568-65	2023	Open single-arm study; 2 weeks (phase 1)	Men/women 18-65 year with schizophrenia (stable, DSM-5)	Ulotaront 50-75mg/d (add-on to current antipsychotic treatment)	22	F-DOPA PET	F-DOPA PET (narratively)

SB: Single-blind; DB: Double-blind; RCT: Randomized controlled trial. The list of eligible studies and a more detailed table of study characteristics can be found in the extended data. *This study was a single-arm neuroimaging study that was included *post-hoc* to provide mechanistic insights of the effects of TAAR1 agonists. **There were usable data only for the adult population.

All of the RCTs had an overall low risk of bias according to RoB2, except for some concerns in one single dose crossover study ^48^, and a high risk in the study of Parkinson’s Disease psychosis due to missing outcome data ( extended data) ^45^.

***Primary outcome***

**Figure 2.**
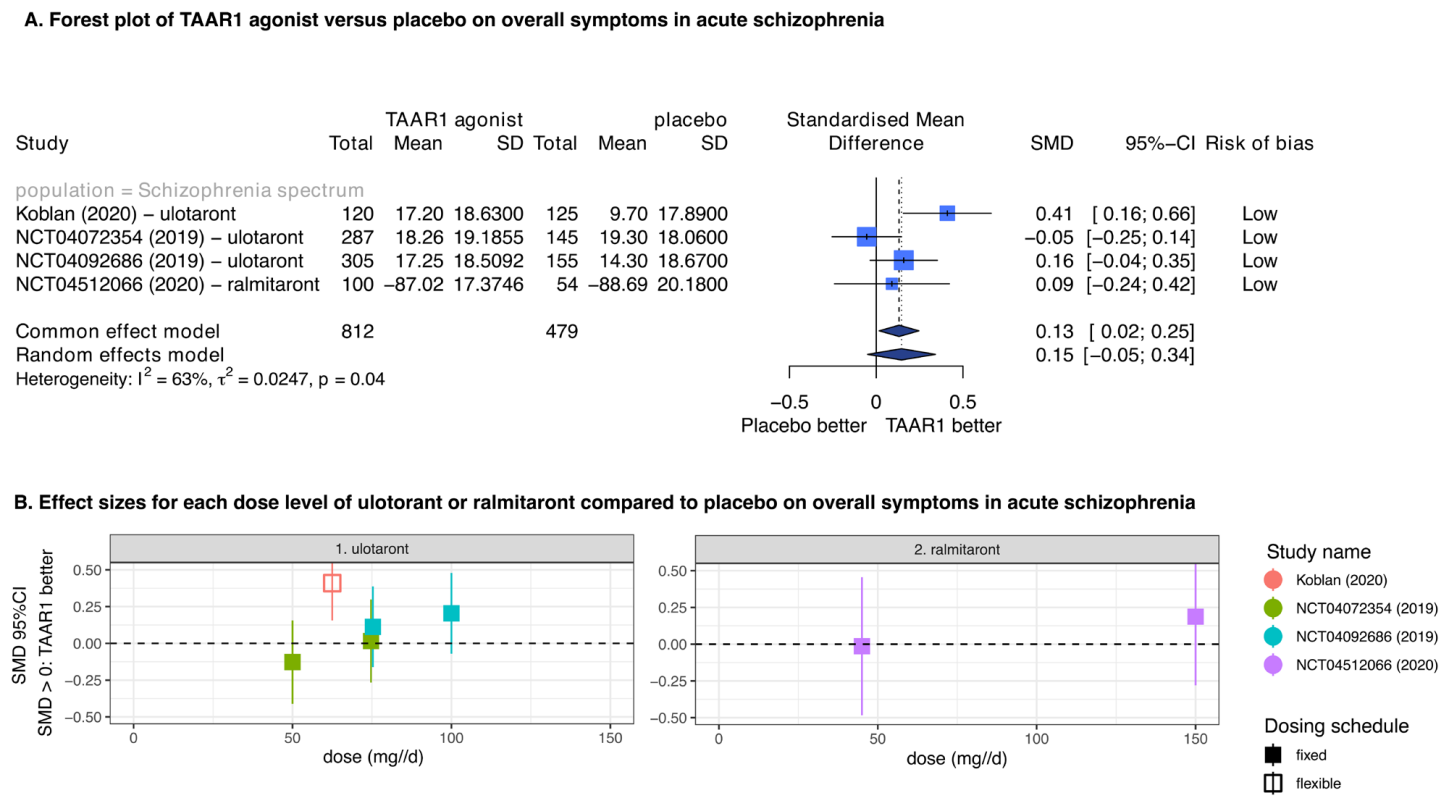
TAAR1 agonists versus placebo on overall symptoms in acute schizophrenia. **A**. Forest plot of TAAR1 agonists versus placebo on overall symptoms in acute schizophrenia. There were available data for ulotaront and ralmitaront. The findings of random- and fixed-effects meta-analyses are presented. The overall risk of bias according to RoB2 is also presented, with all studies having an overall low risk of bias. **B**. Effect sizes for each dose level of ulotaront and ralmitaront compared to placebo on overall symptoms in acute schizophrenia. The findings of each study are presented separately, and the effect sizes were corrected for using the same control in each study by subdividing accordingly the sample size. The study of Koblan *et al.* 2020 was a flexible dosing study, which used of ulotaront between 50 to 75mg/d and its effect size is placed in the middle of the range. SMD: Standardised mean difference, 95%CI: 95% confidence interval.

In participants with acute schizophrenia, TAAR1 agonists showed little difference compared to placebo in improving overall symptoms measured by PANSS total over a treatment of 4–6 weeks (number of studies N=4, number of participants n=1291, SMD=0.15, 95%CI: -0.05 to 0.34) ( Figure 2A). The data were limited for conducting subgroup analyses, with no clear differences between effect sizes for ulotaront and ralmitaront, and no clear indications of dose-response relationships ( Figure 2B). There were concerns of underestimation of the effects of ulotaront due to large placebo response (i.e., observed symptom improvement in the placebo groups) in two of the ulotaront trials ^50, 51, 54^. One RCT found that ralmitaront was less efficacious than the antipsychotic risperidone (n=1, N=156, SMD=-0.53, 95%CI: -0.86 to -0.20) ^52^, and no other study directly compared TAAR1 agonists with antipsychotics.

In participants with Parkinson’s Disease psychosis, one small study found no clear differences between ulotaront and placebo in improving overall symptoms at 6 weeks (n=1, N=37, SMD=-0.28, 95%CI: -0.95 to 0.38) ^45^.

***Secondary outcomes***

**Secondary efficacy outcomes**

The findings on response to treatment were in agreement with those of the primary outcome, showing no clear differences between TAAR1 agonists and placebo ( extended data). There were limited data for other secondary efficacy outcomes including symptom domains.

**Dropouts**

**Figure 3.**
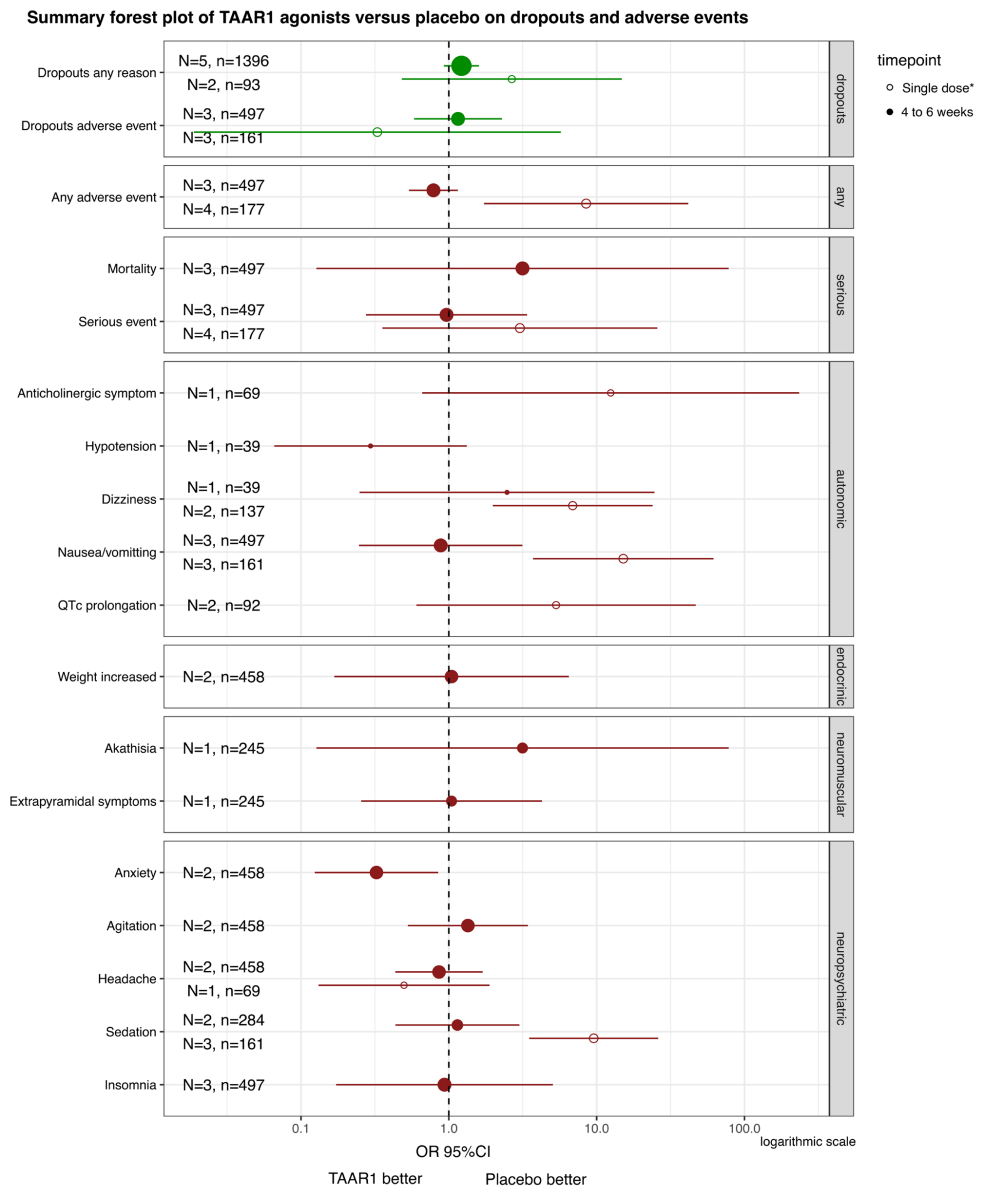
TAAR1 agonists versus placebo on dropouts and adverse events in human studies. Summary forest plot of TAAR1 agonists versus placebo on dropouts and adverse events. The pooled estimates from meta-analyses or effect sizes from single studies are presented for each of the outcomes relevant to dropouts and adverse events, separately for two timepoints with usable data. To ease the interpretation, adverse events were grouped in categories. There were usable data for treatment of ulotaront and ralmitaront for 4 to 6 weeks in acute schizophrenia or Parkinson’s disease psychosis, and after a single dose of ulotaront in participants with schizophrenia, healthy volunteers and narcolepsy-cataplexy. *This time point referred to events experienced after a single dose of ulotaront, except for additional and limited data for any and serious adverse events from the study by Szabo *et al.* 2023 examining treatment of 2 weeks with ulotaront. A logarithmic x-axis was used. N: number of studies; n: number of participants; OR: Odds ratio; 95%CI: 95% confidence interval.

TAAR1 agonists appeared to be associated with more dropouts due to any reason compared to placebo in participants with psychosis over treatment of 4–6 weeks. Still, the effects were small and imprecise (N=5, n=1396, OR=1.22, 95%CI: 0.93, 1.60) ( Figure 3) There was no clear difference between TAAR1 agonists and placebo in dropouts due to adverse events (N=3, n=497; OR=1.15, 95%CI: 0.58, 2.29) ( Figure 3). There was a moderate risk of missing evidence.

**Adverse events**

The data on adverse events were limited, often accompanied by imprecise estimates, and moderate risk of missing evidence or indirectness ( extended data).

TAAR1 agonists did not appear to differ from placebo in adverse events in participants with psychosis at 4–6 weeks, including for serious adverse events, nausea/vomiting, QTc prolongation, weight increase, prolactin elevation, akathisia, extrapyramidal side-effects, sedation and insomnia, with absolute frequencies generally not exceeding 5% ( Figure 3). Moreover, one RCT found that ralmitaront had fewer adverse events of any type than risperidone (OR=0.38, 95%CI: 0.21, 0.68), less nausea/vomiting (OR=0.16, 95%CI: 0.03, 0.84) or weight increase (OR=0.12, 95%CI: 0.02, 0.58) in 214 participants with acute schizophrenia ^52^. A single dose of ulotaront, when compared to placebo, was associated with nausea/vomiting (N=3, n=161, OR=15.14, 95% CI: 3.71, 61.75) and sedation (N=3, n=161, OR=9.55, 95% CI: 3.50, 26.09) in participants with schizophrenia or healthy volunteers, with absolute frequencies approaching 30% of participants ( Figure 3).

**Mechanistic insights**

Two neuroimaging studies ^46, 53^ supported the notion that TAAR1 agonists can regulate dopaminergic signalling in the striatum (more details in extended data). One RCT indicated that a single dose of ulotaront may modulate striatal responses during the anticipatory phase of the Monetary Incentive Delay task using functional magnetic resonance imaging in 96 healthy volunteers ^46^. This task is related to reward processing and served as an indicator of heightened dopaminergic signalling ^46^. Another single-arm trial demonstrated that a 2-week treatment of ulotaront appeared to reduce striatal dopamine synthesis capacity measured with F-DOPA positron emission tomography (PET) (-3.98% from baseline; 95% CI: -8.68%, 0.72%) in 22 clinically stable participants with schizophrenia ^53, 54^. This reduction correlated with the improvement in positive symptoms of psychosis ^53, 54^.

### Findings from non-human studies

***Study description***

**Figure 4.**
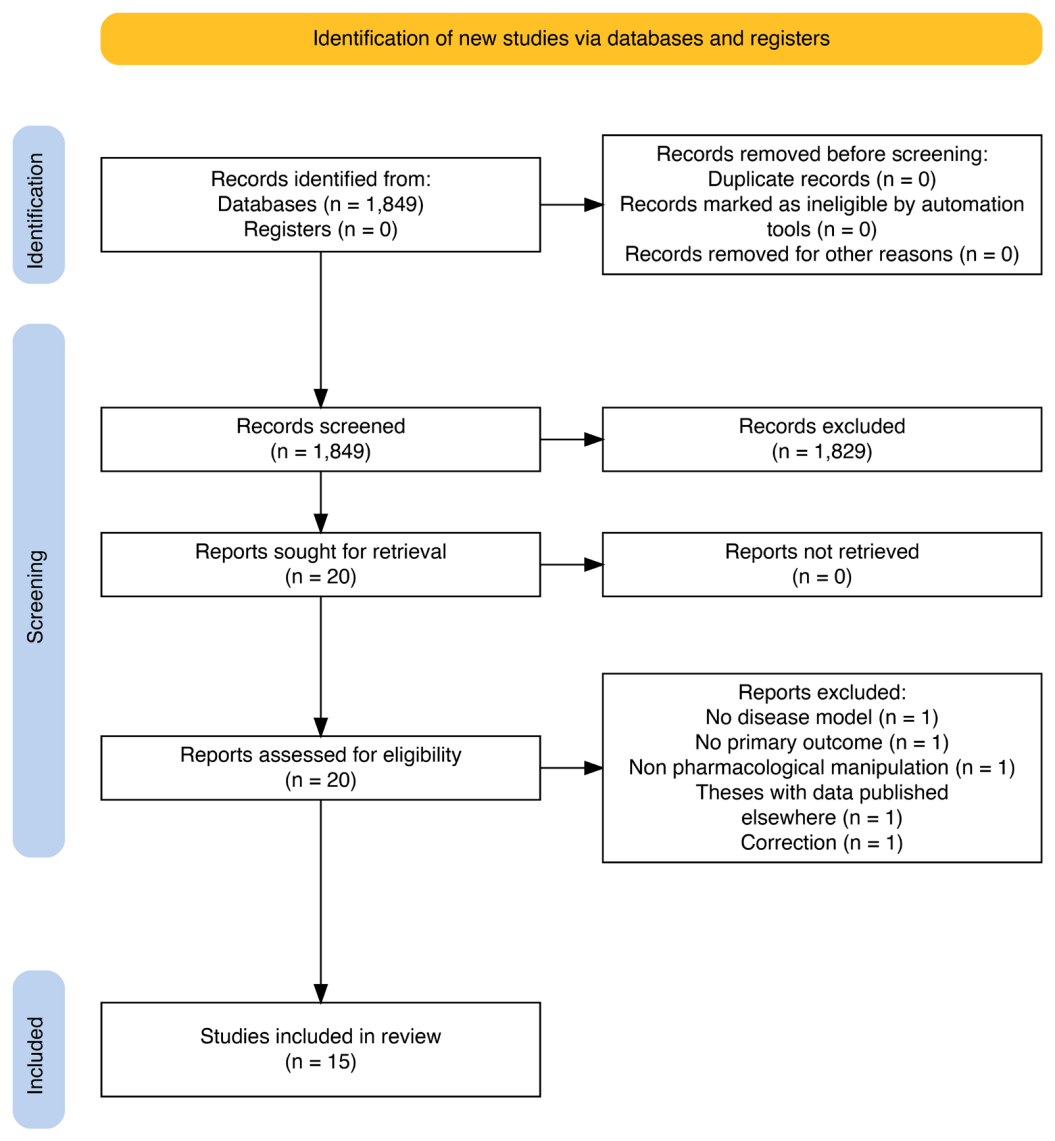
PRISMA flow diagram for study selection of non-human studies.

We screened 1849 titles/abstracts and 20 full texts ( Figure 4), ultimately including 15 studies ^55– 69^ ( Table 2). These studies encompassed multiple experiments involving 38 unique genetic or pharmacological methods to induce psychosis-like behaviours in rodents of various strains and sexes. The studies examined 10 different TAAR1 agonists (AP163, compound 50A, compound 50B, LK000764, RO5073012, RO5166017, RO5203648, RO5256390, RO5263397 and ulotaront), primarily administered as single doses in 84% of the cases, either individually or combined with antipsychotics. TAAR1 agonists were compared with control conditions similar to placebo in clinical trials (e.g., vehicle), and antipsychotics (i.e., aripiprazole, clozapine, olanzapine, and risperidone). The reporting quality according to the modified ARRIVE checklist was generally poor, resulting in unclear risk of bias assessments across most of the studies and the different domains of the SYRCLE’s tool ( extended data).

**Table 2.** Table of characteristics of non-human studies.

Study name	Year	Model	Strain and species	Interventions	Outcomes with usable data
Begni *et al.* 2021 ^55^	2021	Pharmacological	Lister hooded (rat)	Ulotaront; Vehicle	Locomotor activity, Cognition
Cinque *et al.* 2018 ^56^	2018	Genetic	Wistar (rat)	RO5203648; Vehicle	Cognition
Dedic *et al.* 2019 ^57^	2019	Pharmacological	G57BL/6J (mouse); Sprague-Dawley (rat)	Ulotaront; Clozapine; Vehicle	Locomotor activity; Social interaction
Galley *et al.* 2012 ^58^	2012	Pharmacological	Wistar (rat)	RO5073012; Vehicle	Locomotor activity
Kokkinou *et al.* 2021 ^59^	2021	Pharmacological	C67BL/6 (mouse)	Ulotaront; Vehicle	F-DOPA PET
Krasavin *et al.* 2022a ^60^	2022	Pharmacological; Genetic	Wistar (rat)	LK000764; Vehicle	Locomotor activity
Krasavin *et al.* 2022b ^61^	2022	Genetic	Wistar (rat)	AP163; Vehicle	Locomotor activity
Leo *et al.* 2018 ^62^	2018	Genetic	Wistar (rat)	RO5203648; Vehicle	Locomotor activity
Liang *et al.* 2022 ^63^	2022	Pharmacological	ICR (mouse)	Ulotaront; Ulotaront + Olanzapine; Olanzapine; Vehicle	Locomotor activity; Cognition
Revel *et al.* 2011 ^66^	2011	Pharmacological; Genetic	C57BL/6J (mouse); NMRI (mouse)	RO5166017; Vehicle	Locomotor activity; Stereotypy
Revel *et al.* 2012a ^65^	2012	Pharmacological; Genetic	C57BL/6Jx129Sv/J (mouse); C57BL/6J (mouse); Wister (rat)	RO5203648; Vehicle	Locomotor activity
Revel *et al.* 2012b ^64^	2012	Pharmacological	C57BL/6J (mouse)	RO573012; Vehicle	Locomotor activity
Revel 2013 ^69^	2013	Pharmacological	C57BL/6J (mouse); Long-Evans (rat); Not stated (mouse)	RO5256390; RO5263397; RO5256397 + risperidone; Olanzapine; Risperidone; Vehicle	Locomotor activity; Cognition
Saarinen *et al.* 2022 ^68^	2022	Pharmacological	Not stated (mouse)	Ulotaront; Vehicle	Locomotor activity; Prepulse inhibition
Wang *et al.* 2023 ^67^	2023	Pharmacological	C57BL/6J (mouse)	Compound 50A; Compound 50B; Aripiprazole; Risperidone; Vehicle	Locomotor activity

Vehicle: control condition in animal studies like placebo in clinical trials. A more detailed table of study characteristics with the included comparisons of interventions along with the sample size for each outcome can be found in the extended data.

***Primary outcomes***

**Figure 5.**
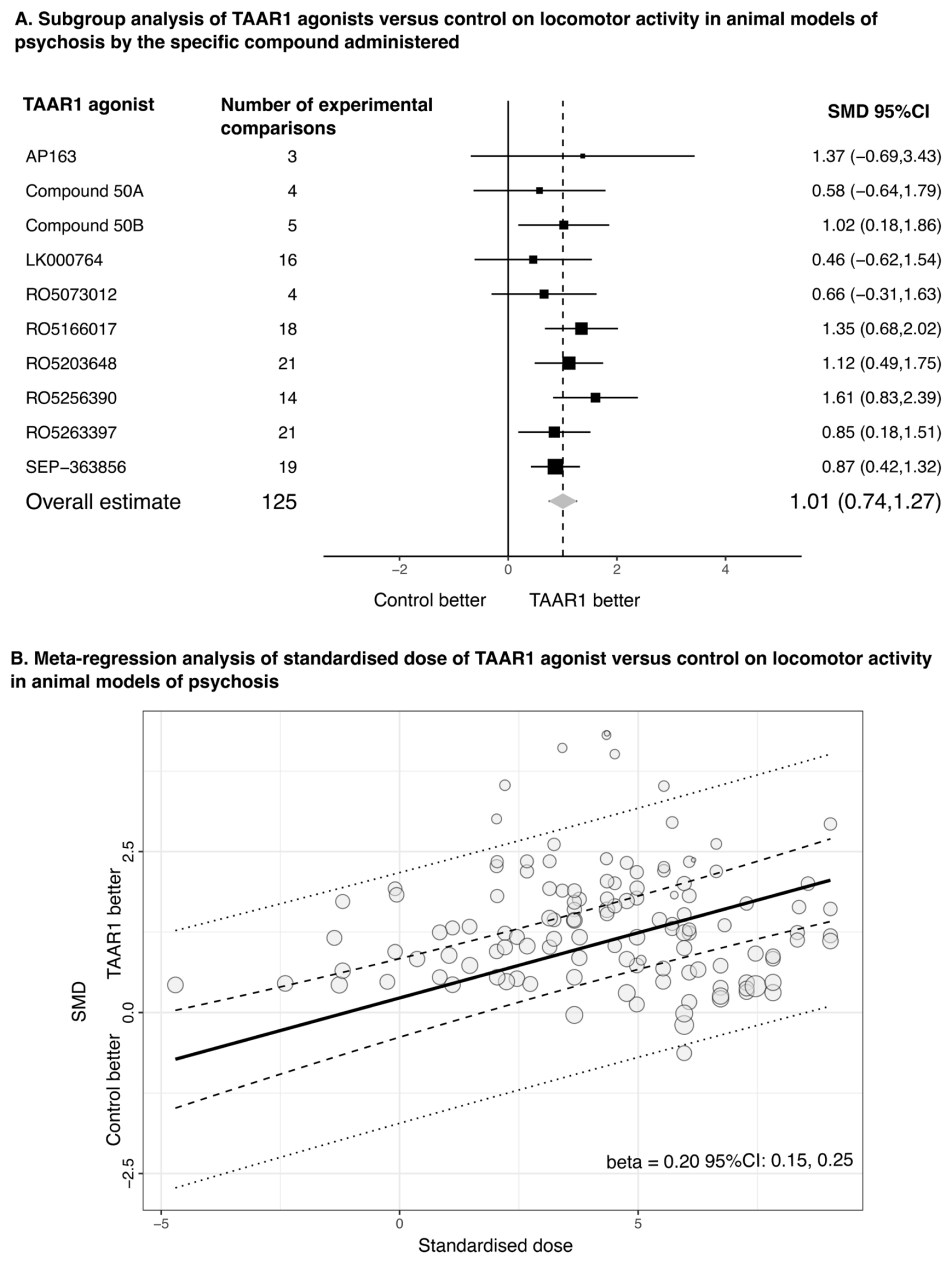
TAAR1 agonists versus control condition for locomotor activity in animal models of psychosis. **A**. Subgroup analysis of TAAR1 agonists versus control condition (e.g., vehicle) for locomotor activity in animal models of psychosis by the specific compound administered. The forest plot presents the overall estimate and the pooled estimates for each of the 10 different TAAR1 agonists by combining data from experimental comparisons. SEP-363856 refers to ulotaront. **B**. Meta-regression analysis of standardized dose of TAAR1 agonists versus control on locomotor activity in animal models of psychosis. A standardised dose was calculated by taking into consideration the drug potency to TAAR1 receptor, with a standardised dose of 0 reflecting 50% and of 1 around 80% of the maximum effect, respectively (see extended data). The dashed lines represent the 95% confidence intervals, and the dotted lines the 95% prediction intervals. The beta for the slope is also presented indicating the change in the magnitude of the effect size per unit change in standardised dose. SMD: Standardized mean difference; 95%CI: 95% confidence intervals.

TAAR1 agonists had overall positive and large effects compared to control conditions on locomotor activity in animal models of psychosis (N=13, number of experiments k=41; SMD=1.01, 95% CI: 0.74, 1.27) with point estimates of SMDs for individual compounds ranging from 0.46 for LK000746 to 1.61 for R05256390 ( Figure 5A). Some heterogeneity was observed, mainly attributed to between-experiment variance (τ ^2^=0.149) and to a smaller degree to between-study variance (τ ^2^=0.054). Higher doses of TAAR1 agonists appeared to be associated with higher efficacy in meta-regression of dose standardized to the drug potency. An increase from doses reflecting 50% of the maximum efficacy to 80% was associated with an average increase in the effect size by 0.20 (95% CI: 0.15, 0.25) ( Figure 5B). However, no dose-response effects were identified for ulotaront (p=0.997). The results were generally robust in sensitivity analysis, but study biases and small-study effects might have exaggerated effect sizes ( extended data).

**Figure 6.**
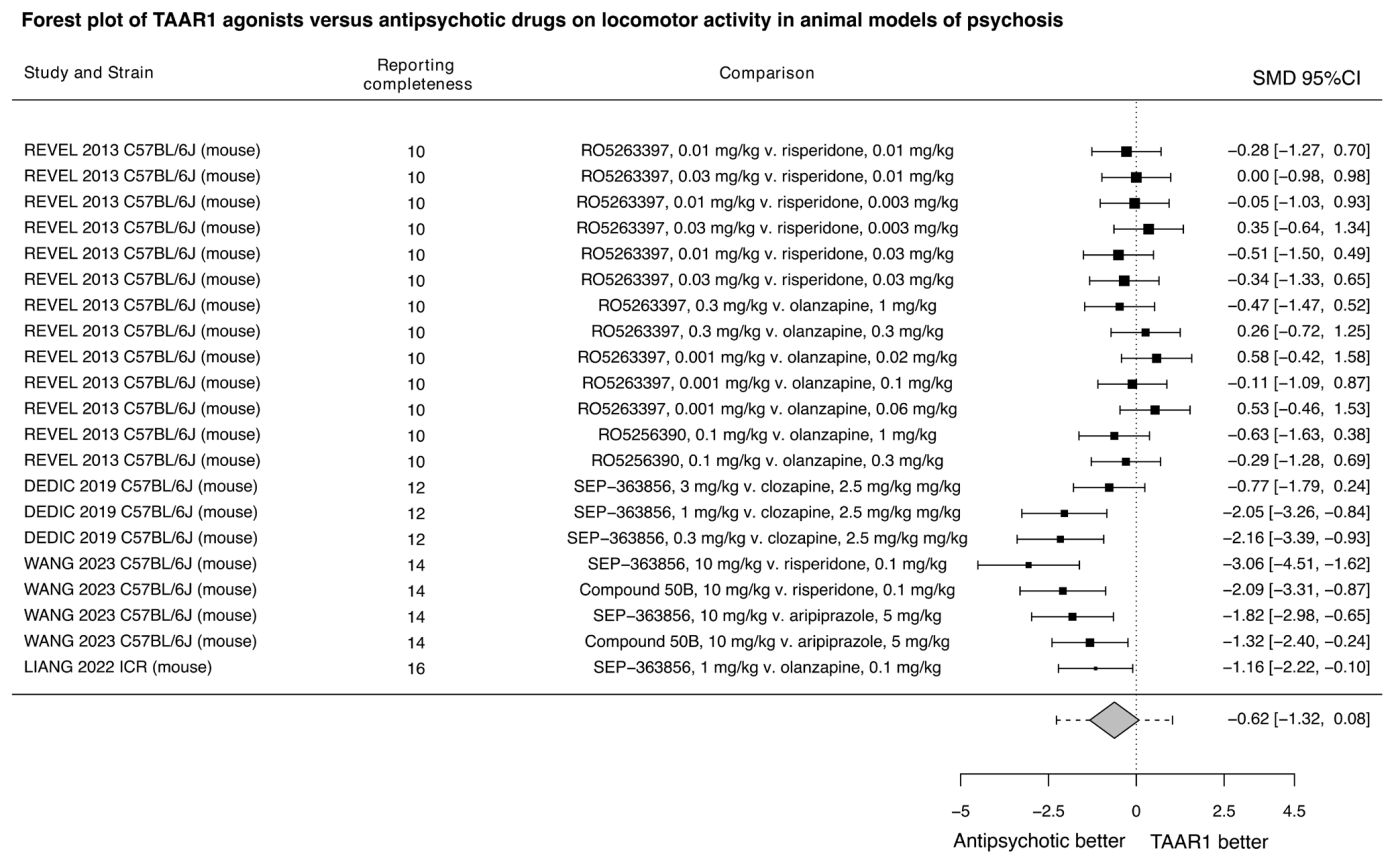
TAAR1 agonists versus already licensed antipsychotics for locomotor activity in animal models of psychosis. Forest plot of TAAR1 agonists versus antipsychotic drugs already licensed for schizophrenia on locomotor activity in animal models of psychosis. The effect sizes for each experimental comparison and the pooled estimate from the multilevel meta-analysis are presented. The dotted lines around the pooled estimate represent the 95% prediction intervals. The reporting completeness according to the modified ARRIVE checklist is also presented. SEP-363856 refers to ulotaront. SMD: Standardised mean difference, 95%CI: 95% confidence interval.

When compared to antipsychotics, TAAR1 agonists alone appeared to be less efficacious in improving locomotor hyperactivity (N=4, k=7, SMD=-0.62, 95% CI: -1.32, 0.08) ( Figure 6). The combination of TAAR1 agonists with antipsychotics may be more efficacious (N=2, SMD=0.90, 95% CI: 0.00, 1.80) ( extended data). Still, the effects were imprecise, and there were insufficient data to explore potential sources of heterogeneity in these comparisons, which may also stem from different TAAR1 agonists and antipsychotics used.

The data for prepulse inhibition impairment, the second primary outcome, came from just one study in animal models of psychosis, so it was not analysed further.

***Secondary outcomes***

**Secondary efficacy outcomes**

There were limited data for secondary efficacy outcomes, with 4 studies reporting data for cognitive function ^55, 56, 63, 69^. There were no clear differences between TAAR1 agonists and control (N=4, k=5, SMD=0.80, 95% CI: -0.30, 1.90) ( extended data). Increasing the dose of TAAR1 agonists from levels reflecting 50% of the maximum efficacy to 80% might reduce efficacy, with the effect size decreasing on average by -0.21 (95% CI: -0.42, 0.00).

**Dropouts**

Dropouts were reported in 2.6% and 3.3% of animals allocated to TAAR1 agonists and control groups, respectively ( extended data).

**Adverse events**

There were no usable data for adverse events in animal models of psychosis.

**Mechanistic insights**

TAAR1 agonists did not appear to have an effect compared to control on locomotor activity in animals subjected to both models of psychosis and TAAR1 knockout (N=2, SMD= -0.02, 95%CI: -1.15, 1.12) ( extended data).

There were no usable data on neurobiological outcomes, except for some indications from one small study that ulotaront may decrease striatal dopamine synthesis capacity measured with F-DOPA PET compared to control in ketamine-treated mice when administered 2 days after completion of the ketamine treatment (SMD = -1.01, 95% CI: -2.04, 0.02) ^59^.

## Discussion

For the first time, we combined a quantitative synthesis of data from human and non-human sources of evidence regarding the efficacy, tolerability, and mechanism of action of TAAR1 agonists in treating psychosis, which goes beyond previous reviews ^6, 9, 70– 73^.

### Summary of findings and interpretation

TAAR1 agonists may be less efficacious than dopamine D _2 _receptor blocking drugs licensed for schizophrenia, yet the results are inconclusive. In clinical trials, ulotaront and ralmitaront exhibited an average effect size of 0.15 compared to placebo in acute schizophrenia, which is smaller than other antipsychotics (ranging from 0.24 to 0.89) ^1^. However, large placebo responses in the two ulotaront phase III trials raised assay sensitivity concerns ^50, 51, 54^. This could be partly attributed to recruitment challenges during the COVID-19 pandemic, and a post-hoc analysis using participants recruited before the pandemic reported efficacy comparable to that of the phase II trial ( Figure 2) ^7, 54^. Limited data were available for direct comparisons between the two drug classes, with ralmitaront being less efficacious than risperidone in one trial ^52^. Additional insights were provided by animal studies that examined more compounds and revealed that TAAR1 agonists may be less efficacious when directly compared to dopamine D _2_ receptor blocking antipsychotics. Still, TAAR1 agonists demonstrated dose-dependent positive effects compared to control groups in these studies. This may suggest that the doses in human studies might have been insufficient (up to ulotaront 100mg/d, ralmitaront 150mg/d). Nevertheless, no dose-response relationship was identified for ulotaront up to 10mg/kg/day in rodents, roughly corresponding to 75–150mg/day in humans ^74^.

In terms of tolerability, evidence from clinical trials suggested that TAAR1 agonists may have a relatively benign side-effect profile, as ulotaront and ralmitaront did not show clear differences from placebo in adverse events over 4–6 weeks. However, single-dose trials of ulotaront reported sedation and nausea/vomiting, indicating possible transient effects.

From a mechanistic standpoint, limited but consistent evidence from F-DOPA PET human and animal studies suggested that ulotaront may alleviate presynaptic dopamine dysregulation in the striatum ^53, 59^, a central factor in the development of psychosis symptoms ^75^, in contrast to dopamine D _2_ receptor blocking drugs ^76, 77^. Additionally, the findings from animal models of psychosis indicated that TAAR1 agonists potentially exert their beneficial effects through TAAR1-dependent inference with dopaminergic signalling. However, sedative effects may also play a role potentially explaining the opposite direction of the dose-response relationships for locomotor activity and cognitive function.

### Limitations

The review had some limitations. First, data were available for a limited number of TAAR1 agonists, and only ulotaront and ralmitaront in human studies, which may not be generalisable for this entire drug category. Recent studies have shed further light on the structure and function of TAAR1, paving the way on developing new drugs with unique pharmacological properties, such as functional selectivity, that may yield different findings ^10, 11, 78– 81^. Additionally, clinical trials were primarily conducted in the United States of America and Eastern Europe with limited representation of countries from the global south.

Second, there was missing evidence. We could not include all data from unpublished human studies, including those involving adolescents ^50^, predominant negative symptoms (terminated early due to inefficacy in interim analysis) ^82^, the maintenance phase ^83^, and from the ulotaront phase III trials ^50, 51^ for which we could only locate data on overall symptoms from a conference abstract ^54^. Additionally, there was the possibility that relevant animal studies were unpublished or missed, as indicated by small-study effects. Although we comprehensively searched multiple databases, conventional screening of titles/abstracts underperform in identifying non-human studies ^84^. Thus, alongside conducting the first iteration of this review, we developed systematic online living evidence summaries (SOLES) ^84^ for preclinical psychosis research ( Psychosis-SOLES) ^85^ that use automated tools to facilitate study identification. To further improve future review updates, we used the current search terms and data extraction template for human data as input to the development of associated ontology classes and relationships ^86^, which would support data searching and data-driven algorithms for enrichment and inference.

Third, we focused on RCTs and excluded uncontrolled and observational human studies. Uncontrolled studies mainly addressed pharmacokinetics (see excluded studies in extended data), except for the six-month open-label extension of the ulotaront phase II trial ^7^, which highlighted its long-term efficacy and benign side-effect profile ^87^. Observational studies, including genetic and post-mortem studies, have suggested an association between dysregulated TAAR1 activity and schizophrenia ^6, 88– 90^. In non-human studies, we focused on *in vivo* animal models of psychosis and excluded those not subjected to such models. However, TAAR1 agonists can still be evaluated in naïve animals or other specific models ^10, 57, 91^. For example, ulotaront appeared to have a similar efficacy in improving prepulse inhibition (co-primary outcome in the synthesis of non-human data) in both naïve animals ^57^ and models of psychosis ^68^. Yet, meta-analysis for this outcome was not feasible in this review iteration due to limited data from only one study using animal models of psychosis ^68^. We excluded *in vitro* studies despite insights they could offer on the mechanism of action of TAAR1 agonists ^6^. Synthesizing their data would be challenging due to the limited established systematic review methods ^92, 93^.

Finally, we were unable to explore heterogeneity due to the small number of human studies. Individual participant data are necessary for refined examination of potential participant-level characteristics, including age, sex, chronicity of illness, baseline severity, and understanding potential reasons for the small effect sizes observed in ulotaront phase III trials ^50, 51^. Moreover, differences in the findings of human and non-human studies underscored the challenges of modelling a complex and subjective condition like psychosis in animals, with unclear and limited validity ^4, 94, 95^. This emphasises the need for systematic examination and improvement of the translatability of animal models of psychosis.

### Reflections on the co-production of the review

GALENOS experiential advisors ^12, 13^, co-produced the plain language summary, contributed to protocol design and interpretation of the findings. They also improved the presentation of review methodologies and findings to the wider public. Emphasis was placed on contextualising the effects of new medications with those of already licensed antipsychotics, especially regarding symptoms of psychosis, such as hallucinations and delusions, and adverse events like weight gain.

This review is the first of a series of living systematic reviews within the GALENOS project ^12^, establishing the basis for inclusion of experiential advisors in future reviews and development of a more systemic co-production process. Honest discussions addressed the nature of engagement of experiential advisors in academic setting (e.g. working towards deadlines, in large teams across multiple countries), the need for responsive two-way communication, and the challenges faced. The aim was for experiential advisors to be involved as equal partners in review teams, underscoring the importance of training and supporting experiential advisors and researchers, and of levelling the field so that participants understand well enough the content of the work being carried out. Consequently, alongside with conducting the review, we co-produced guidance on how best to include experiential advisors in the GALENOS reviews ^14^.

## Conclusions

Evidence from both human and animal studies suggested that current TAAR1 agonists may offer smaller benefits in improving symptoms of psychosis compared to dopamine D _2_ receptor blocking drugs already licensed for schizophrenia. However, the findings were inconclusive due to possible biases, and there was some disconnect between human and animal studies, highlighting the need for further improving the translatability of animal models in the context of psychosis. Limited evidence also suggested that TAAR1 agonists may regulate presynaptic dopaminergic signalling and have a relatively benign side-effect profile. The field of TAAR1 research remains active, with ongoing drug development. Thus, the TAAR1 mechanism of action still holds promise for the treatment of psychosis, with emerging evidence expected in the near future. Future iterations of this living systematic review and meta-analysis will aim to incorporate these new developments.

## Ethics and consent

Ethical approval and consent were not required.

## Abbreviations

5-HT _1A_: Serotonin 5-HT _1A_ receptor; 95%CI: 95% confidence intervals; ARRIVE: Animal Research Reporting of *In Vivo* Experiments; d: Day; D _2_: Dopamine D _2_ receptor; EPPI-Reviewer: Evidence for Policy, and Practice Information and Coordinating Centre - Reviewer; F-DOPA: Fluorodopa; GALENOS: Global Alliance for Living Evidence on Anxiety, Depression, and Psychosis; GRIPP: Guidance for Reporting Involvement of Patients and the Public; k: Number of experiments; kg: Kilogram; mg: Milligram; N: Number of studies; n: Number of participants; NMD: Normalised mean differences; OR: Odds Ratio; PANSS: Positive and Negative Syndrome Scale; PET: Positron emission tomography; PRISMA: Preferred Reporting Items for Systematic Reviews and Meta-Analyses; PRISMA-S: PRISMA Statement for Reporting Literature Searches; RCT: Randomised controlled trials; RoB2: Risk of Bias 2 tool; SEP-363856: Ulotaront; SMD: Standardised mean differences; SyRF: Systematic Review Facility; TAAR1: Trace amine-associated receptor 1

## Data Availability

The data for this article consists of bibliographic references, which are included in the References section. Aggregated data and R code for the analysis can be found in the GitHub repositories for the project: Human studies: https://github.com/galenos-project/LSR3_taar1_H. The archived aggregated data and analysis code at time of publication, https://doi.org/10.5281/zenodo.10890206 ^43^ Non-human studies: https://github.com/galenos-project/LSR3_taar1_A. The archived aggregated data and analysis code at time of publication, https://doi.org/10.5281/zenodo.10890218 ^44^. Underlying data are available under the terms of the Creative Commons Attribution 4.0 International license (CC-BY 4.0). Open Science Framework: Trace amine-associated receptor 1 (TAAR1) agonists for psychosis: protocol for a living systematic review and meta-analysis of human and non-human studies, https://doi.org/10.17605/OSF.IO/TDMAU ^96^. This project contains the following extended data: The report of the findings for the human studies published in Rpubs: https://rpubs.com/sksiafis/LSR3_TAAR1_H The report of the findings for the non-human studies published in Rpubs: https://rpubs.com/VirginiaChiocchia/LSR3_TAAR1_A Extended data are available under the terms of the Creative Commons Attribution 4.0 International license (CC-BY 4.0). Open Science Framework: PRISMA 2020, PRISMA-S and GRIPP2 checklists can be found in Open Science Framework, https://doi.org/10.17605/OSF.IO/TDMAU ^96^. Completed checklists for the corresponding reporting guidelines are available under the terms of the Creative Commons Attribution 4.0 International license (CC-BY 4.0).
