# Supplementary material for: Trace amine-associated receptor 1 (TAAR1) agonism for psychosis: a living systematic review and meta-analysis of human and non-human data
Source: Wellcome Open Res. 2024 Apr 11;9:182. [Version 1] doi: 10.12688/wellcomeopenres.21302.1 (PMC11258611; doi:10.12688/wellcomeopenres.21302.1)
Supplement: Peer review history

# Reviewer report: Reviewer response for version 1

**DOI:** 10.21956/wellcomeopenres.23562.r85393

**Published:** 2024-07-19

**Authors:** Gavin P Reynolds

**Affiliations:** 1 Sheffield Hallam University, Sheffield, England, UK

This article addresses the role of TAAR1 agonist/partial agonist drugs in the treatment of psychosis. It has a rigorous approach and is co-produced with "experiential advisors"; I also believe it is unique in its approach of synthesising data from both human and non-human studies. In the light of recent clinical reports, its findings are perhaps unsurprising - that TAAR1 agonists have a "relatively benign" profile in terms of adverse effects but may be less effective than current antipsychotic treatments.  

 Previous reviewers have made some suggestions/comments but there are no comments from the authors or indication of how the review may have been modified in response. There have been some modifications "post hoc" including a focus on RCTs rather than including all experimental human studies; this deserves emphasising more clearly when ref 18 is mentioned, rather than just referring to "extended data". The protocol provides a useful list of research questions; it would have been valuable to have those repeated here.

 Most definitely the living review format is a valuable one in this rapidly moving field, although on current clinical evidence its lifetime may be limited!

 Given the statements in recent publications suggesting a possible efficacy of ulotaront on negative symptoms, it would have been valuable to have seen this addressed explicitly in the clinical and animal data, if only to say there is inadequate/inconsistent evidence.

 Notwithstanding the involvement of the experiential advisors, there is some excessive use of jargon - one example being that of "triangulation" which means different things to different people; a few words of explanation as to what is considered in a triangulation meeting would be helpful.

 While the assessment of dropouts is important in clinical studies, the use of this term in the animal studies is confusing since it inevitably relates to technical issues rather than factors such as tolerability. 

 It would have been valuable to have included in the animal forest plots a word indicating the psychosis model used for each of the studies.

 The authors provide a useful limitations section.

Are the rationale for, and objectives of, the Systematic Review clearly stated?

Yes

Is the statistical analysis and its interpretation appropriate?

I cannot comment. A qualified statistician is required.

If this is a Living Systematic Review, is the ‘living’ method appropriate and is the search schedule clearly defined and justified? (‘Living Systematic Review’ or a variation of this term should be included in the title.)

Yes

Are sufficient details of the methods and analysis provided to allow replication by others?

Yes

Are the conclusions drawn adequately supported by the results presented in the review?

Yes

Reviewer Expertise:

Psychopharmacology and biological psychiatry of schizophrenia

I confirm that I have read this submission and believe that I have an appropriate level of expertise to confirm that it is of an acceptable scientific standard.

# Reviewer report: Reviewer response for version 1

**DOI:** 10.21956/wellcomeopenres.23562.r80883

**Published:** 2024-07-17

**Authors:** Pramod Nair

**Affiliations:** 1 Flinders University, Adelaide, South Australia, Australia

The manuscript by Siafis *et al.*, provides a comprehensive systematic review and meta-analysis of human and non-human data on trace amine-associated receptor 1 (TAAR1) agonism for psychosis. This is an important ‘living’ systematic review examining the effects of TAAR1, which aims to provide regular updates on this emerging pharmacological target in the field of psychosis and neuropsychiatric disorders.

 The current study includes 9 clinical RCT studies, investigating the effects of TAAR1 agonists on a total of 1683 individuals (average age: 32.9 years, 69.8% men, 30.2% women), and 15 preclinical studies conducted on different laboratory psychotic mice models.

 According to this study, TAAR1 agonists may be less effective than dopamine D2 receptor antagonists already available for treating schizophrenia. However, this interpretation is considered preliminary and inconclusive due to the limited number of drugs examined, the lack of longer-term data, the disconnect between human and animal studies, publication bias, *etc*. There is also limited evidence that suggests TAAR1 agonists may promote presynaptic dopaminergic signalling while being relatively safe. Overall, the authors provided a widespread overview and effectively described the current state of TAAR1 agonists for the treatment of psychosis. Considering the ongoing development of TAAR1 agonists and their potential to treat neuropsychiatric disorders, the article and any future iterations carry a great deal of importance.

Minor errors need to be addressed:

 Figure 1:

 The screening section mentions n=165 studies. Under the section “Findings from human studies” on page 12, Study description, Line 1 shows 166 full texts. Which one is true?

 Page 13: Primary outcome:

 Lines 3-4 say participants n= 1291. In table 1, total sample size of all 4 studies is 1457. Not clear. Line 14 says participants N = 156. This number doesn’t match the numbers in Figure 2A or Table 1. If exclusions are made it needs to be mentioned in the text/legends.

 In Table 1:

 According to clinicaltrails.gov, NCT04092686 ( https://clinicaltrials.gov/study/NCT04092686) had 464 (actual enrolments), 462 (subjects). Same with NCT04072354 ( https://clinicaltrials.gov/study/NCT04072354?cond=NCT04072354&rank=1), 463 (actual enrolment), 435 subjects. Which numbers are considered? Actual enrolment or treatment subjects? The same extends to all other studies mentioned here.

 The primary outcome, Second paragraph: “…. Ralmitaront was less efficacious than the antipsychotic ….”. Which figure is showing this?

 Figure 2B: units on the x-axis show “mg//d” Is it meant to be mg/d?

 Figure 3:

 A legend is required for the colours, what do green circles represent? What is red?

 The figure needs refining. Can the grey cross lines be removed? The labels need to be aligned properly. For example: see the “dropouts” section, the line is sitting on top of the text. Keep consistent font size and colours.

 Some dots seem to be bigger than others. What does it mean? Should be clarified in the legends.

 Figure 5:

 Standardised dosage. Units?

 Some squares seem to be bigger than others. What does it mean?

 Suggestion: All figures comparing “placebo/antipsychotic better or TAAR1 better”, will be easier for the reader if they are colour-coded. Red when placebo/antipsychotic is better and green when TAAR1 is better or vice versa. Removing the minor and major gridlines from all figures may help present cleaner figures.

Are the rationale for, and objectives of, the Systematic Review clearly stated?

Yes

Is the statistical analysis and its interpretation appropriate?

I cannot comment. A qualified statistician is required.

If this is a Living Systematic Review, is the ‘living’ method appropriate and is the search schedule clearly defined and justified? (‘Living Systematic Review’ or a variation of this term should be included in the title.)

Yes

Are sufficient details of the methods and analysis provided to allow replication by others?

Yes

Are the conclusions drawn adequately supported by the results presented in the review?

Yes

Reviewer Expertise:

Schizophrenia pharmacology, neuropsychiatric disorders, drug design and discovery, development of small molecular therapeutics.

I confirm that I have read this submission and believe that I have an appropriate level of expertise to confirm that it is of an acceptable scientific standard.

# Reviewer report: Reviewer response for version 1

**DOI:** 10.21956/wellcomeopenres.23562.r85389

**Published:** 2024-06-20

**Authors:** Keiko Ikemoto

**Affiliations:** 1 The University of Tokyo, Tokyo, Japan

The authors try to review efficacy of TAAR1 for psychosis combined with meta-analysis of human and non-human data.  In humans, data of clinical trials of ulotaront and ralmitaront were available, which showed mild effectiveness of TAAR1

 agonists for treatment of psychosis.

 The authors mention placebo effects, possible influence of COVID-19 pandemic on results of clinical trials, and expectancy of TAAR1 agonists.

 This is a timely review.  

    

 Previous post-mortem brain studies by Ikemoto et al (Ref -1). have shown the reduction of TAAR1-ligand neurons (D-neurons) in the nucleus accumbens, an antipsychotic acting site, of patients with schizophrenia (with long disease duration), which is a backgrounded datum for expectancy of TAAR1 medicinal chemistry. Please cite this study and discuss the results based on the post-mortem brain study. 

Are the rationale for, and objectives of, the Systematic Review clearly stated?

Yes

Is the statistical analysis and its interpretation appropriate?

I cannot comment. A qualified statistician is required.

If this is a Living Systematic Review, is the ‘living’ method appropriate and is the search schedule clearly defined and justified? (‘Living Systematic Review’ or a variation of this term should be included in the title.)

Yes

Are sufficient details of the methods and analysis provided to allow replication by others?

Yes

Are the conclusions drawn adequately supported by the results presented in the review?

Yes

Reviewer Expertise:

clinical psychiatry, histochemistry

I confirm that I have read this submission and believe that I have an appropriate level of expertise to confirm that it is of an acceptable scientific standard.

## References

[1] : D-Neuron, Ligand Neuron of Trace Amine-Associated Receptor 1 (TAAR1): Key of Novel Non-D2 Receptor-Binding Antipsychotics. *Journal of Biotechnology and Biomedicine* .2024;07(01) : 10.26502/jbb.2642-91280123 10.26502/jbb.2642-91280123
